# 3-Ethyl­sulfanyl-2,5-diphenyl-1-benzofuran

**DOI:** 10.1107/S1600536810028308

**Published:** 2010-07-21

**Authors:** Hong Dae Choi, Pil Ja Seo, Byeng Wha Son, Uk Lee

**Affiliations:** a Department of Chemistry, Dongeui University, San 24 Kaya-dong Busanjin-gu, Busan 614-714, Republic of Korea; b Department of Chemistry, Pukyong National University, 599-1 Daeyeon 3-dong, Nam-gu, Busan 608-737, Republic of Korea

## Abstract

In the title compound, C_22_H_18_OS, the 2-phenyl ring is rotated out of the benzofuran plane, making a dihedral angle of 29.18 (6)°. The dihedral angle between the 5-phenyl ring and the benzofuran plane is 20.42 (5)°. In the crystal structure, mol­ecules are linked by weak inter­molecular C—H⋯π inter­actions.

## Related literature

For the crystal structures of similar 3-alkyl­sulfanyl-2,5-diaryl-1-benzofuran derivatives, see: Choi, *et al.* (2006 ▶, 2010 ▶). For the pharmacological activity of benzofuran compounds, see: Aslam *et al.* (2006 ▶); Galal *et al.* (2009 ▶); Khan *et al.* (2005 ▶). For natural products with benzofuran rings, see: Akgul & Anil (2003 ▶); Soekamto *et al.* (2003 ▶). 
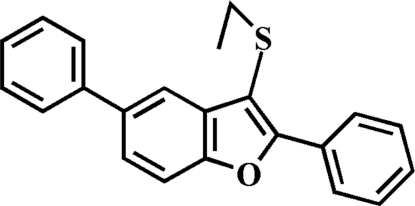


## Experimental

### 

#### Crystal data

C_22_H_18_OS
*M* *_r_* = 330.42
Monoclinic, 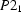

*a* = 10.4968 (3) Å
*b* = 7.2025 (2) Å
*c* = 12.0783 (3) Å
β = 112.474 (1)°
*V* = 843.81 (4) Å^3^
*Z* = 2
Mo *K*α radiation
μ = 0.20 mm^−1^
*T* = 174 K
0.24 × 0.20 × 0.18 mm

#### Data collection

Bruker SMART APEXII CCD diffractometer
Absorption correction: multi-scan (*SADABS*; Bruker, 2009 ▶) *T*_min_ = 0.954, *T*_max_ = 0.966
7930 measured reflections
3380 independent reflections
3229 reflections with *I* > 2σ(*I*)
*R*_int_ = 0.028

#### Refinement

*R*[*F* ^2^ > 2σ(*F* ^2^)] = 0.031
*wR*(*F* ^2^) = 0.080
*S* = 1.06
3380 reflections
218 parameters
1 restraint
H-atom parameters constrained
Δρ_max_ = 0.20 e Å^−3^
Δρ_min_ = −0.24 e Å^−3^
Absolute structure: Flack (1983 ▶), 1271 Friedel pairs
Flack parameter: 0.05 (6)

### 

Data collection: *APEX2* (Bruker, 2009 ▶); cell refinement: *SAINT* (Bruker, 2009 ▶); data reduction: *SAINT*; program(s) used to solve structure: *SHELXS97* (Sheldrick, 2008 ▶); program(s) used to refine structure: *SHELXL97* (Sheldrick, 2008 ▶); molecular graphics: *ORTEP-3* (Farrugia, 1997 ▶) and *DIAMOND* (Brandenburg, 1998 ▶); software used to prepare material for publication: *SHELXL97*.

## Supplementary Material

Crystal structure: contains datablocks global, I. DOI: 10.1107/S1600536810028308/cs2130sup1.cif

Structure factors: contains datablocks I. DOI: 10.1107/S1600536810028308/cs2130Isup2.hkl

Additional supplementary materials:  crystallographic information; 3D view; checkCIF report

## Figures and Tables

**Table 1:** Hydrogen-bond geometry (Å, °) *Cg*1 and *Cg*2 are the centroids of the C9–C14 (5-phen­yl) and C15–C20 (2-phen­yl) rings, respectively.

*D*—H⋯*A*	*D*—H	H⋯*A*	*D*⋯*A*	*D*—H⋯*A*
C10—H10⋯*Cg*1^i^	0.95	2.73	3.592 (3)	152
C14—H14⋯*Cg*2^ii^	0.95	2.79	3.549 (3)	138

Symmetry codes: (i) 
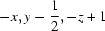
; (ii) 
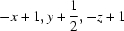
.

**Fig. 1.**
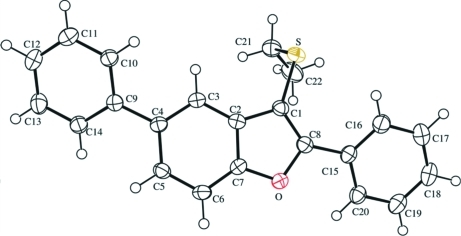
The molecular structure of the title compound with the atom numbering scheme. Displacement ellipsoids are drawn at the 50% probability level. H atoms are presented as a small circles of arbitrary radius.

**Fig. 2.**
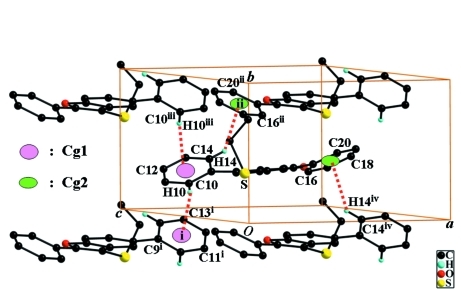
C—H···π interactions (dotted lines) in the crystal structure of the title compound. Cg denotes the ring centroid. [Symmetry codes: (i) - x, y - 1/2, - z + 1; (ii) - x + 1, y + 1/2, - z + 1; (iii) - x, y + 1/2, - z + 1; (iv) - x + 1, y - 1/2, - z + 1.]

## supplementary crystallographic information

### Comment

The compounds containing benzofuran skeleton show interesting pharmacological properties such as antifungal (Aslam *et al.*., 2006), antitumor and antiviral (Galal *et al.*., 2009), antimicrobial (Khan *et al.*., 2005) activity. These compounds occur in nature (Akgul & Anil, 2003; Soekamto *et al.*., 2003). As a part of our ongoing studies of the effect of side chain substituents on the solid state structures of 3-alkylsulfanyl-2,5-diaryl-1-benzofuran analogues (Choi *et al.*., 2006, 2010), we report the crystal structure of the title compound (Fig. 1).

The title compound crystallizes in the monoclinic space group P2_1_. The benzofuran unit is essentially planar, with a mean deviation of 0.020 (1) Å from the least-squares plane defined by the nine constituent atoms. In the molecule, the benzofuran plane makes dihedral angles of 29.18 (6) and 20.42 (5)° with the 2-phenyl ring and the 5-phenyl ring, respectively. The crystal packing (Fig. 2) is stabilized by weak intermolecular C—H···π interactions; the first one between the 5-phenyl H atom and the 5-phenyl ring of an adjacent molecule, with a C10—H10···Cg1^i^, and the second one between the 5-phenyl H atom and the 2-phenyl ring of a neighbouring molecule, with a C14—H14···Cg2^ii^, respectively (Table 1, Cg1 and Cg2 are the centroids of the C9–C14 phenyl ring and the C15–C20 phenyl ring, respectively, for symmetry operators see Fig. 2 legend).

### Experimental

Zinc chloride (300 mg, 2.2 mmol) was added to a stirred solution of 4-phenylphenol (375 mg, 2.2 mmol) and 2-chloro-2-(ethylsulfanyl)acetophenone (472 mg, 2.2 mmol) in dichloromethane (30 mL) at room temperature, and stirring was continued at the same temperature for 1hr. The reaction was quenched by the addition of water and the organic layer separated, dried over magnesium sulfate, filtered and concentrated at reduced pressure. The residue was purified by column chromatography (carbon tetrachloride) to afford the title compound as a colorless solid [yield 46%, m.p. 368–369 K; *R*_f_ = 0.51 (carbon tetrachloride)]. Single crystals suitable for X-ray diffraction were prepared by evaporation of a solution of the title compound in acetone at room temperature.

### Refinement

All H atoms were positioned geometrically and refined using a riding model, with C—H = 0.95 Å for aryl, 0.95 Å for methylene and 0.99 Å for methyl H atoms. *U*_iso_(H) = 1.2*U*_eq_(C) for aryl and methylene, 1.5*U*_eq_(C) for methyl H atoms.

### Crystal data

**Table:**

C_22_H_18_OS	*F*(000) = 348
*M**_r_* = 330.42	*D*_x_ = 1.300 Mg m^−^^3^
Monoclinic, *P*2_1_	Mo *K*α radiation, λ = 0.71073 Å
Hall symbol: P 2yb	Cell parameters from 5754 reflections
*a* = 10.4968 (3) Å	θ = 2.2–27.6°
*b* = 7.2025 (2) Å	µ = 0.20 mm^−^^1^
*c* = 12.0783 (3) Å	*T* = 174 K
β = 112.474 (1)°	Block, colourless
*V* = 843.81 (4) Å^3^	0.24 × 0.20 × 0.18 mm
*Z* = 2	

### Data collection

**Table:**

Bruker SMART APEXII CCD diffractometer	3380 independent reflections
Radiation source: rotating anode	3229 reflections with *I* > 2σ(*I*)
graphite multilayer	*R*_int_ = 0.028
Detector resolution: 10.0 pixels mm^-1^	θ_max_ = 27.6°, θ_min_ = 1.8°
φ and ω scans	*h* = −13→13
Absorption correction: multi-scan (*SADABS*; Bruker, 2009)	*k* = −8→9
*T*_min_ = 0.954, *T*_max_ = 0.966	*l* = −15→15
7930 measured reflections	

### Refinement

**Table:**

Refinement on *F*^2^	Secondary atom site location: difference Fourier map
Least-squares matrix: full	Hydrogen site location: difference Fourier map
*R*[*F*^2^ > 2σ(*F*^2^)] = 0.031	H-atom parameters constrained
*wR*(*F*^2^) = 0.080	*w* = 1/[σ^2^(*F*_o_^2^) + (0.0418*P*)^2^ + 0.1018*P*] where *P* = (*F*_o_^2^ + 2*F*_c_^2^)/3
*S* = 1.06	(Δ/σ)_max_ < 0.001
3380 reflections	Δρ_max_ = 0.20 e Å^−^^3^
218 parameters	Δρ_min_ = −0.24 e Å^−^^3^
1 restraint	Absolute structure: Flack (1983), 1271 Friedel pairs
Primary atom site location: structure-invariant direct methods	Flack parameter: 0.05 (6)

### Special details

**Table:**

Geometry. All esds (except the esd in the dihedral angle between two l.s. planes) are estimated using the full covariance matrix. The cell esds are taken into account individually in the estimation of esds in distances, angles and torsion angles; correlations between esds in cell parameters are only used when they are defined by crystal symmetry. An approximate (isotropic) treatment of cell esds is used for estimating esds involving l.s. planes.
Refinement. Refinement of F^2^ against ALL reflections. The weighted R-factor wR and goodness of fit S are based on F^2^, conventional R-factors R are based on F, with F set to zero for negative F^2^. The threshold expression of F^2^ > 2sigma(F^2^) is used only for calculating R-factors(gt) etc. and is not relevant to the choice of reflections for refinement. R-factors based on F^2^ are statistically about twice as large as those based on F, and R- factors based on ALL data will be even larger.

### Fractional atomic coordinates and isotropic or equivalent isotropic displacement parameters (Å^2^)

**Table:**

	*x*	*y*	*z*	*U*_iso_*/*U*_eq_	
S	0.52254 (4)	0.25661 (7)	0.85171 (3)	0.03269 (11)	
O	0.64105 (10)	0.32731 (17)	0.57860 (9)	0.0280 (2)	
C1	0.54722 (15)	0.2879 (2)	0.71715 (13)	0.0257 (3)	
C2	0.43709 (15)	0.2982 (2)	0.60132 (13)	0.0247 (3)	
C3	0.29352 (15)	0.2969 (2)	0.55943 (13)	0.0252 (3)	
H3	0.2485	0.2815	0.6138	0.030*	
C4	0.21667 (15)	0.3182 (2)	0.43754 (13)	0.0247 (3)	
C5	0.28644 (16)	0.3366 (2)	0.35838 (14)	0.0278 (3)	
H5	0.2336	0.3477	0.2749	0.033*	
C6	0.42891 (16)	0.3391 (3)	0.39822 (14)	0.0293 (3)	
H6	0.4747	0.3519	0.3444	0.035*	
C7	0.50068 (15)	0.3221 (2)	0.51984 (14)	0.0259 (3)	
C8	0.66776 (15)	0.3084 (2)	0.69972 (14)	0.0266 (3)	
C9	0.06340 (15)	0.3277 (2)	0.39132 (13)	0.0242 (3)	
C10	−0.00910 (15)	0.2388 (3)	0.45240 (14)	0.0275 (3)	
H10	0.0395	0.1659	0.5212	0.033*	
C11	−0.15161 (15)	0.2563 (3)	0.41335 (15)	0.0323 (3)	
H11	−0.1996	0.1959	0.4558	0.039*	
C12	−0.22321 (17)	0.3606 (3)	0.31357 (17)	0.0350 (4)	
H12	−0.3203	0.3741	0.2879	0.042*	
C13	−0.15343 (18)	0.4461 (3)	0.25046 (16)	0.0365 (4)	
H13	−0.2031	0.5160	0.1805	0.044*	
C14	−0.01146 (17)	0.4299 (3)	0.28911 (15)	0.0301 (4)	
H14	0.0354	0.4893	0.2454	0.036*	
C15	0.81365 (15)	0.3226 (2)	0.77699 (14)	0.0274 (3)	
C16	0.86570 (16)	0.2563 (3)	0.89490 (15)	0.0353 (4)	
H16	0.8056	0.1992	0.9269	0.042*	
C17	1.00454 (18)	0.2741 (3)	0.96477 (16)	0.0404 (4)	
H17	1.0393	0.2287	1.0447	0.048*	
C18	1.09330 (19)	0.3569 (3)	0.91986 (19)	0.0394 (4)	
H18	1.1884	0.3690	0.9687	0.047*	
C19	1.04281 (18)	0.4221 (3)	0.80334 (18)	0.0374 (4)	
H19	1.1037	0.4793	0.7723	0.045*	
C20	0.90512 (17)	0.4049 (2)	0.73196 (16)	0.0313 (4)	
H20	0.8718	0.4491	0.6518	0.038*	
C21	0.45785 (19)	0.4872 (3)	0.86615 (17)	0.0391 (4)	
H21A	0.3815	0.5199	0.7900	0.047*	
H21B	0.4203	0.4841	0.9299	0.047*	
C22	0.5674 (2)	0.6348 (3)	0.89573 (19)	0.0489 (5)	
H22A	0.6086	0.6339	0.8354	0.073*	
H22B	0.6389	0.6100	0.9749	0.073*	
H22C	0.5260	0.7566	0.8962	0.073*	

### Atomic displacement parameters (Å^2^)

**Table:**

	*U*^11^	*U*^22^	*U*^33^	*U*^12^	*U*^13^	*U*^23^
S	0.0348 (2)	0.0388 (2)	0.02657 (19)	−0.00208 (19)	0.01411 (16)	0.00605 (19)
O	0.0247 (5)	0.0343 (6)	0.0265 (5)	−0.0002 (5)	0.0113 (4)	−0.0006 (5)
C1	0.0280 (7)	0.0243 (9)	0.0257 (7)	0.0003 (6)	0.0112 (6)	0.0029 (6)
C2	0.0298 (7)	0.0220 (9)	0.0242 (7)	−0.0011 (6)	0.0124 (6)	−0.0006 (6)
C3	0.0266 (7)	0.0251 (9)	0.0268 (7)	−0.0011 (6)	0.0135 (6)	−0.0002 (6)
C4	0.0264 (7)	0.0222 (8)	0.0275 (7)	−0.0027 (6)	0.0124 (6)	−0.0017 (6)
C5	0.0306 (8)	0.0316 (9)	0.0221 (7)	−0.0020 (7)	0.0110 (6)	−0.0007 (7)
C6	0.0322 (8)	0.0334 (9)	0.0278 (8)	−0.0022 (7)	0.0175 (7)	−0.0015 (7)
C7	0.0237 (7)	0.0268 (8)	0.0288 (8)	−0.0006 (6)	0.0120 (6)	−0.0013 (7)
C8	0.0298 (7)	0.0240 (8)	0.0264 (7)	0.0028 (6)	0.0113 (6)	0.0008 (6)
C9	0.0268 (7)	0.0211 (7)	0.0244 (7)	−0.0009 (6)	0.0096 (6)	−0.0038 (6)
C10	0.0276 (7)	0.0260 (8)	0.0293 (7)	−0.0020 (7)	0.0115 (6)	−0.0007 (7)
C11	0.0306 (8)	0.0299 (8)	0.0402 (8)	−0.0039 (8)	0.0177 (7)	−0.0030 (9)
C12	0.0238 (8)	0.0355 (10)	0.0425 (10)	−0.0022 (7)	0.0090 (7)	−0.0037 (8)
C13	0.0309 (9)	0.0362 (10)	0.0352 (9)	0.0012 (7)	0.0047 (8)	0.0045 (8)
C14	0.0300 (9)	0.0293 (9)	0.0303 (8)	−0.0038 (7)	0.0107 (7)	0.0020 (7)
C15	0.0261 (7)	0.0229 (8)	0.0331 (8)	0.0021 (7)	0.0112 (7)	−0.0017 (7)
C16	0.0308 (8)	0.0367 (9)	0.0378 (8)	0.0019 (8)	0.0123 (7)	0.0029 (9)
C17	0.0362 (9)	0.0421 (12)	0.0347 (8)	0.0047 (9)	0.0046 (7)	0.0023 (9)
C18	0.0260 (8)	0.0352 (10)	0.0491 (11)	0.0029 (7)	0.0054 (8)	−0.0021 (9)
C19	0.0300 (9)	0.0327 (10)	0.0504 (11)	0.0002 (7)	0.0163 (8)	0.0014 (9)
C20	0.0284 (8)	0.0275 (9)	0.0375 (9)	0.0024 (7)	0.0119 (7)	0.0026 (7)
C21	0.0408 (10)	0.0484 (12)	0.0320 (9)	0.0039 (9)	0.0181 (8)	−0.0019 (8)
C22	0.0638 (13)	0.0459 (12)	0.0350 (10)	−0.0056 (10)	0.0168 (10)	−0.0048 (9)

### Geometric parameters (Å, °)

**Table:**

S—C1	1.755 (1)	C12—C13	1.387 (3)
S—C21	1.828 (2)	C12—H12	0.9500
O—C7	1.370 (2)	C13—C14	1.386 (2)
O—C8	1.387 (2)	C13—H13	0.9500
C1—C8	1.368 (2)	C14—H14	0.9500
C1—C2	1.436 (2)	C15—C16	1.400 (2)
C2—C3	1.394 (2)	C15—C20	1.403 (2)
C2—C7	1.395 (2)	C16—C17	1.383 (2)
C3—C4	1.391 (2)	C16—H16	0.9500
C3—H3	0.9500	C17—C18	1.380 (3)
C4—C5	1.416 (2)	C17—H17	0.9500
C4—C9	1.489 (2)	C18—C19	1.383 (3)
C5—C6	1.385 (2)	C18—H18	0.9500
C5—H5	0.9500	C19—C20	1.377 (2)
C6—C7	1.376 (2)	C19—H19	0.9500
C6—H6	0.9500	C20—H20	0.9500
C8—C15	1.461 (2)	C21—C22	1.505 (3)
C9—C14	1.394 (2)	C21—H21A	0.9900
C9—C10	1.401 (2)	C21—H21B	0.9900
C10—C11	1.392 (2)	C22—H22A	0.9800
C10—H10	0.9500	C22—H22B	0.9800
C11—C12	1.375 (3)	C22—H22C	0.9800
C11—H11	0.9500		
			
C1—S—C21	99.40 (8)	C13—C12—H12	120.1
C7—O—C8	106.70 (11)	C14—C13—C12	120.22 (17)
C8—C1—C2	106.98 (13)	C14—C13—H13	119.9
C8—C1—S	128.97 (12)	C12—C13—H13	119.9
C2—C1—S	124.05 (11)	C13—C14—C9	120.83 (15)
C3—C2—C7	119.15 (14)	C13—C14—H14	119.6
C3—C2—C1	135.14 (13)	C9—C14—H14	119.6
C7—C2—C1	105.64 (13)	C16—C15—C20	118.66 (15)
C4—C3—C2	119.50 (12)	C16—C15—C8	122.16 (14)
C4—C3—H3	120.2	C20—C15—C8	119.18 (15)
C2—C3—H3	120.2	C17—C16—C15	119.99 (16)
C3—C4—C5	119.01 (13)	C17—C16—H16	120.0
C3—C4—C9	120.55 (12)	C15—C16—H16	120.0
C5—C4—C9	120.42 (14)	C18—C17—C16	120.83 (17)
C6—C5—C4	122.36 (14)	C18—C17—H17	119.6
C6—C5—H5	118.8	C16—C17—H17	119.6
C4—C5—H5	118.8	C17—C18—C19	119.55 (17)
C7—C6—C5	116.58 (13)	C17—C18—H18	120.2
C7—C6—H6	121.7	C19—C18—H18	120.2
C5—C6—H6	121.7	C20—C19—C18	120.60 (16)
O—C7—C6	126.30 (13)	C20—C19—H19	119.7
O—C7—C2	110.36 (13)	C18—C19—H19	119.7
C6—C7—C2	123.34 (14)	C19—C20—C15	120.36 (16)
C1—C8—O	110.31 (13)	C19—C20—H20	119.8
C1—C8—C15	135.63 (14)	C15—C20—H20	119.8
O—C8—C15	114.01 (12)	C22—C21—S	112.73 (14)
C14—C9—C10	118.17 (14)	C22—C21—H21A	109.0
C14—C9—C4	121.24 (13)	S—C21—H21A	109.0
C10—C9—C4	120.55 (14)	C22—C21—H21B	109.0
C11—C10—C9	120.68 (16)	S—C21—H21B	109.0
C11—C10—H10	119.7	H21A—C21—H21B	107.8
C9—C10—H10	119.7	C21—C22—H22A	109.5
C12—C11—C10	120.21 (15)	C21—C22—H22B	109.5
C12—C11—H11	119.9	H22A—C22—H22B	109.5
C10—C11—H11	119.9	C21—C22—H22C	109.5
C11—C12—C13	119.87 (15)	H22A—C22—H22C	109.5
C11—C12—H12	120.1	H22B—C22—H22C	109.5
			
C21—S—C1—C8	106.15 (17)	C7—O—C8—C15	−176.39 (13)
C21—S—C1—C2	−73.27 (15)	C3—C4—C9—C14	149.59 (16)
C8—C1—C2—C3	−176.13 (17)	C5—C4—C9—C14	−28.4 (2)
S—C1—C2—C3	3.4 (3)	C3—C4—C9—C10	−28.1 (2)
C8—C1—C2—C7	0.84 (18)	C5—C4—C9—C10	153.88 (17)
S—C1—C2—C7	−179.64 (12)	C14—C9—C10—C11	−1.5 (2)
C7—C2—C3—C4	0.4 (2)	C4—C9—C10—C11	176.25 (16)
C1—C2—C3—C4	177.06 (16)	C9—C10—C11—C12	0.3 (3)
C2—C3—C4—C5	1.4 (2)	C10—C11—C12—C13	1.1 (3)
C2—C3—C4—C9	−176.65 (14)	C11—C12—C13—C14	−1.3 (3)
C3—C4—C5—C6	−1.8 (3)	C12—C13—C14—C9	0.2 (3)
C9—C4—C5—C6	176.31 (15)	C10—C9—C14—C13	1.2 (3)
C4—C5—C6—C7	0.2 (3)	C4—C9—C14—C13	−176.49 (16)
C8—O—C7—C6	178.86 (16)	C1—C8—C15—C16	21.8 (3)
C8—O—C7—C2	−0.62 (18)	O—C8—C15—C16	−161.40 (16)
C5—C6—C7—O	−177.68 (15)	C1—C8—C15—C20	−158.25 (19)
C5—C6—C7—C2	1.7 (3)	O—C8—C15—C20	18.5 (2)
C3—C2—C7—O	177.43 (13)	C20—C15—C16—C17	0.5 (3)
C1—C2—C7—O	−0.13 (18)	C8—C15—C16—C17	−179.59 (17)
C3—C2—C7—C6	−2.1 (3)	C15—C16—C17—C18	0.1 (3)
C1—C2—C7—C6	−179.63 (16)	C16—C17—C18—C19	−0.3 (3)
C2—C1—C8—O	−1.26 (18)	C17—C18—C19—C20	−0.1 (3)
S—C1—C8—O	179.25 (12)	C18—C19—C20—C15	0.7 (3)
C2—C1—C8—C15	175.57 (17)	C16—C15—C20—C19	−0.9 (3)
S—C1—C8—C15	−3.9 (3)	C8—C15—C20—C19	179.21 (16)
C7—O—C8—C1	1.18 (17)	C1—S—C21—C22	−70.50 (15)

### Hydrogen-bond geometry (Å, °)

**Table:**

Cg1 and Cg2 are the centroids of the C9–C14 (5-phenyl) and C15–C20 (2-phenyl) rings, respectively.

**Table:**

*D*—H···*A*	*D*—H	H···*A*	*D*···*A*	*D*—H···*A*
C10—H10···Cg1^i^	0.95	2.73	3.592 (3)	152
C14—H14···Cg2^ii^	0.95	2.79	3.549 (3)	138

Symmetry codes: (i) −*x*, *y*−1/2, −*z*+1; (ii) −*x*+1, *y*+1/2, −*z*+1.
